# Altered Glycosylation in the Aging Heart

**DOI:** 10.3389/fmolb.2021.673044

**Published:** 2021-05-28

**Authors:** Patricia Franzka, Lynn Krüger, Mona K. Schurig, Maja Olecka, Steve Hoffmann, Véronique Blanchard, Christian A. Hübner

**Affiliations:** ^1^ Institute of Human Genetics, University Hospital Jena, Friedrich Schiller University, Jena, Germany; ^2^ Institute of Laboratory Medicine, Clinical Chemistry and Pathobiochemistry, Humboldt-Universität zu Berlin, and Berlin Institute of Health, Charité-Universitätsmedizin Berlin, Corporate Member of Freie Universität Berlin, Berlin, Germany; ^3^ Department of Biology, Chemistry and Pharmacy, Freie Universität Berlin, Berlin, Germany; ^4^ Hoffmann Research Group, Leibniz-Institute on Aging–Fritz-Lipmann-Institute, Jena, Germany

**Keywords:** cardiac aging, post-translational modifications, glycosylation, cardiac glycoproteome, mannosylation

## Abstract

Cardiovascular disease is one of the leading causes of death in developed countries. Because the incidence increases exponentially in the aging population, aging is a major risk factor for cardiovascular disease. Cardiac hypertrophy, fibrosis and inflammation are typical hallmarks of the aged heart. The molecular mechanisms, however, are poorly understood. Because glycosylation is one of the most common post-translational protein modifications and can affect biological properties and functions of proteins, we here provide the first analysis of the cardiac glycoproteome of mice at different ages. Western blot as well as MALDI-TOF based glycome analysis suggest that high-mannose *N*-glycans increase with age. In agreement, we found an age-related regulation of GMPPB, the enzyme, which facilitates the supply of the sugar-donor GDP-mannose. Glycoprotein pull-downs from heart lysates of young, middle-aged and old mice in combination with quantitative mass spectrometry bolster widespread alterations of the cardiac glycoproteome. Major hits are glycoproteins related to the extracellular matrix and Ca^2+^-binding proteins of the endoplasmic reticulum. We propose that changes in the heart glycoproteome likely contribute to the age-related functional decline of the cardiovascular system.

## Introduction

Aging is associated with the progressive deterioration of the structure and function of the heart and is a dominant risk factor for cardiovascular diseases, the leading cause of death in Western populations. Although the phenotypes of cardiac aging has been well characterized, the molecular mechanisms of cardiac aging are largely unknown. Cardiac aging is characterized by hypertrophy, diastolic dysfunction (Downes et al., 1989; Lakatta, 2003), cellular senescence and inflammation, oxidative stress, mitochondrial dysfunction (Judge et al., 2005) and extracellular matrix (ECM) remodeling (Ouzounian et al., 2008). The increase in the number of senescent cardiomyocytes during aging is associated with the secretion of proinflammatory cytokines, proteases and insoluble ECM components, which promote inflammation and cell death (Wang and Shah, 2015; Shimizu and Minamino, 2019). Main ECM proteins are collagen, elastin, fibronectin, and laminin*,* which have both structural and adhesive functions (Burgess et al., 2001) and are highly glycosylated.

Glycosylation is the most common post-translational modification of proteins and lipids. It is relevant for the majority of plasma membrane and secreted proteins. The glycosylation status of proteins can affect their stability and conformation (Shental-Bechor and Levy, 2008; Traini et al., 2017; Breloy and Hanisch, 2018). Thus, glycosylation plays a prominent role in many biological processes including cell-to-cell communication, cell-matrix interaction, adhesion, protein targeting and folding, viral or bacterial infection, cancer and aging (Banerjee et al., 2017; Traini et al., 2017; Breloy and Hanisch, 2018). While changes in protein glycosylation in the diseased heart are well established (Fülöp et al., 2007; Montpetit et al., 2009; Deng et al., 2016; Nagai-Okatani and Minamino, 2016; Marques-Da-Silva et al., 2017), it is yet unclear whether changes in protein glycosylation may contribute to cardiac aging. Systematic studies on the glycoproteome of the aging cardiac muscle are so far missing.

Here, we show for the first time that the murine cardiac glycoproteome changes during aging. More specifically, we found an increased incorporation of mannose residues into carbohydrate chains. In accordance with increased mannosylation, the abundance of the enzyme facilitating the supply of the sugar donor GDP-mannose, GDP-mannose-pyrophosphorylase-B (GMPPB), increases with age.

## Materials and Methods

Experiments were performed in a C57BL/6 background. For analyses, only male mice were used. Mice were housed in a 12 h light/dark cycle and had access to mouse chow ad libitum (9% fat, 24% protein, 67% carbohydrates including 1% of free mannose). Treatment cohorts were fed with nominally mannose-free food, in which free mannose had been replaced by sucrose. End points were termination of treatment and morbidity for mice in accordance with Institutional Animal Care and Use Committee guidelines. Experiments were conducted blind. Figure legends include details of replicates used to generate data sets. All animal experiments were approved by the “Thüringer Landesamt für Lebensmittelsicherheit und Verbraucherschutz (TLLV)”. Animal numbers are stated in the figure legends.

## Histology and Immunohistochemistry

Mice were sacrificed and fresh heart tissue was immediately frozen in 30% sucrose-OCT (Sakura) in liquid nitrogen and stored at −80°C until further use. Heart tissue was cryo-sectioned into 4 µm thick sections in a cryostat chamber (Kryostar NX70, Thermo Scientific) at −25°C for the specimen and −18°C for the knife.

Immunofluorescence stainings were performed in Shandon chambers (Thermo Scientific). Sections were fixed in 4% paraformaldehyde in 1x phosphate-buffered saline (PBS) for 15 min and rinsed with 1xPBS 3 times. 0.25% (v/v) Triton-X in 1xPBS was used to permeabilize cells. After blocking with 5% normal goat serum in 0.25% (v/v) Triton-X in 1xPBS for 1 h, primary antibodies were applied overnight at 4°C in 0.25% (v/v) Triton-X in 1xPBS: rabbit anti-laminin (Abcam) 1:200, mouse anti-α-actinin (Abcam) 1:200, anti-mouse IgM paucimannose (gift of Rüdiger Horstkorte, Halle) 1:20. Sections were washed with 1xPBS and corresponding secondary antibodies (Invitrogen) were incubated in a 1:1,000 dilution in 1xPBS for 2 h at room temperature (RT). Nuclei were stained with DAPI 1:10,000 (Hoechst, Invitrogen). Sections were mounted with Fluoromount-G (Southern Biotech). Images were taken with a Zeiss LSM880 Airyscan confocal microscope. Z-projections with average intensities that were processed with *ImageJ* are shown. Acquisition parameters and image processing were kept constant.

For measuring the mean cardiomyocyte diameter cross-sectioned cells and longitudinal-sectioned cells were separately evaluated. The mean diameter is defined as the minimal distance between two laminin stained cell borders at the nucleus height.

For measuring the sarcomere length and Z-disc height, α-actinin stained sections were used. The sarcomere length is the horizontal distance between two α-actinin bands. The Z-disc height is defined as the length of one α-actinin band.

For Picro Sirius red stainings sections were stained with hematoxylin (Sigma-Aldrich) for 10 min and afterwards rinsed in running tap water. After washing sections were stained in 0.5% (w/v) Picro Sirius red solution (Sigma Aldrich) for 1 h, then washed in acidified water and mounted with Entellan (Merck). Images were captured with a petrographic microscope AxioImager Z.2 (Zeiss) and further analyzed by *ImageJ*: 15 random fields of view were manually selected and imaged at an objective lens magnification of 20×. In *ImageJ* images were converted to RGB stacks providing grayscale pictures for the red, green and blue channel separately. For the first analysis, the intensities of each channel were measured. For the second analysis, the threshold was adjusted to 20–255 and the measure tool was used to measure the threshold area (% area).

## Protein Isolation From Tissue

Mice were sacrificed and heart tissue was immediately frozen in liquid nitrogen and stored at -80°C until further use. Tissue lysates were prepared with the Ultra-Turrax T8 tissue homogenizer (IKA-WERKE) in TBS-buffer (20 mM Tris, 150 mM NaCl, 1% (v/v) TritonX-100, complete protease inhibitor and complete phosphatase inhibitor (Roche)). After sonication, homogenates were spun down at 16,900 g to remove nuclei and insoluble debris. The supernatant was stored at −80°C until further use.

## Glycome Analysis of Heart Lysates

Mice were sacrificed and heart tissue was immediately frozen in liquid nitrogen and stored at −80°C until further use. Tissue lysates were prepared with the Ultra-Turrax T8 tissue homogenizer (IKA-WERKE) in TBS-buffer (20 mM Tris, 150 mM NaCl, 1% (v/v) TritonX-100, complete protease inhibitor and complete phosphatase inhibitor (Roche)). After sonication, homogenates were spun down at 16,900 g to remove nuclei and insoluble debris.

Prior to *N*-glycan analysis, total heart protein lysates were filtered using 10 kDa Amicon filters (Merck Millipore, Ireland). Afterwards, aliquots corresponding to 200 μg of protein were dissolved in PBS pH 6.5 (250 mM NaH_2_PO_4_, 250 mM Na_2_HPO_4_) and SDS was added to a final concentration of 1% (w/v). Proteins were denatured at 95°C for 5 min. The buffer concentration was adjusted to 160 mM with water and Ipegal at a final concentration of 1% (w/v). Next, *N*-glycan release was performed using 1 U of PNGase F (N-Zyme Scientifics). *N*-glycans were subsequently purified, permethylated and measured by MALDI-TOF mass spectrometry as described earlier (Biskup et al., 2013). The spectra were acquired in *m/z* 1,000–5,000 region in the positive ion mode [M + Na]^+^. For every spectrum acquisition, 10,000 shots were collected. The spectra were acquired at 100 Hz frequency. Detector gain was set up to 1,638 V and the analog offset was 51 mV. Processing of spectra was performed with Flexanalysis (Bruker Daltonics, Bremen, Germany). Glycan structures were assigned using Glycoworkbench (Damerell et al., 2015) and relative areas were calculated from the MALDI-TOF spectra and are presented in Supplementary Table S1. In order to have a comprehensive comparison with our lectin data, *N*-glycans were first grouped by type, namely high-mannose (Man_3_GlcNAc_2_-Man_9_GlcNAc_2_) or complex-type (carrying antennae that extend the core and are initiated by GlcNAc on each antennae). Secondly, we grouped complex-type structures according to the glycosylation traits fucosylation (all structures carrying fucose) and sialylation (all structures carrying sialic acid (NeuGc or NeuAc)). For each glycosylation trait, we summed up the relative areas of the glycan structures of relevance (see Supplementary Table S1). As the intensity of fucosylated *N*-glycan structures was too low to perform MALDI-TOF/TOF fragmentation, *N*-glycan structures were assigned from known biological pathways.

## Western Blot

Proteins were denatured at 90°C for 5 min in Laemmli buffer. After separation by SDS-PAGE (8% polyacrylamide gels) proteins were transferred onto 0.45 µm PVDF membranes (Whatman) at 290 mA for 100 min. Membranes were blocked in 2% BSA for 1 h at RT and incubated with primary antibodies at appropriate dilutions in tris-buffered saline supplemented with 0.1% tween (TBS-T) overnight at 4°C. The following primary antibodies were used: rabbit anti-GMPPA (Proteintech) 1:500, rabbit anti-GMPPB (Proteintech) 1:500, rabbit anti-GAPDH (Santa Cruz) 1:1,000, rabbit anti-nidogen (Abcam) 1:500, rabbit anti-talin-1 (Proteintech) 1:1,000, mouse anti-alpha-actinin (Abcam) 1:500, rabbit anti-beta1-integrin (Abcam) 1:1,000, rabbit anti-calsequestrin (Proteintech) 1:1,000, rabbit anti-calnexin (Sigma) 1:500, rabbit anti-calreticulin (Cell Signaling) 1:500, self-made antibodies mouse anti-oligomannose (6–9 terminal mannose residues), and mouse anti-paucimannose (3 terminal mannose residues) 1:50 (gift of Rüdiger Horstkorte, Halle). Membranes were washed in TBS-T and primary antibodies were detected with horseradish peroxidase-conjugated secondary antibodies in an appropriate dilution. Following secondary antibodies were used: donkey anti-rabbit IgG-HRP (Amersham) 1:4,000, donkey anti-mouse IgG (Amersham) 1:4,000, goat anti-mouse IgM (Invitrogen) 1:4,000. To detect glycans we used the following lectins: biotin-PNA (Peanut agglutinin: Galβ1-3GalNAcα1-R, R = residue) (Vector labs), biotin-RCAI (Ricinus communis agglutinin I: terminal beta 1,4-linked Gal), biotin-SNAI (Sambucus nigra agglutinin isolectin I: Neu5Ac/Gc-α(2,6)-Gal-β(1,4)-GlcNAc-R), biotin-WGA (Wheat germ agglutinin: GlcNAc, Neu5Ac/Gc), biotin MAL (Maackia amurensis agglutinin: Neu5Ac/Gc-α(2,3)-Gal-β(1,4)-GlcNAc-R) (gifts from Dr. Christian Thiel), which were detected with horseradish peroxidase-conjugated streptavidin (Sigma) in a 1:10,000 dilution. Detection was performed with the SuperSignal Western Blot Enhancer Kit (Thermo Fisher Scientific). The quantification of bands was done with ImageJ: Protein bands were selected and their intensity quantified. For background correction, an area of the same size below or above the protein band was selected and the respective intensity subtracted from the band intensities. Intensities were either normalized to GAPDH or Coomassie blue stained PVDF membranes. Band intensities of (glyco)-proteins of a high molecular weight were either normalized to GAPDH, vinculin or Coomassie blue stained PVDF membranes. GAPDH and vinculin abundance did not change with age.

Coomassie blue staining of PVDF membranes was performed after protein detection. For Coomassie blue staining of transferred proteins, PVDF membranes were fixed for 3 min (10% acetic acid, 40% EtOH), stained in Coomassie blue solution (0.1% Brilliant Blue R (Serva), 45% EtOH, 10% acetic acid) for 5 min, destained (10% acetic acid, 20–40% EtOH), rinsed in H_2_O and imaged.

## Sugar Measurements

Blood was taken from unfasted mice and incubated on ice for 15 min. Samples were centrifuged for 10 min at 4°C and 4,000 g. Sugars were measured in the supernatant with the D-mannose, D-fructose, D-glucose kit following manufacturer’s instructions (Megazyme, K-MANGL).

## Glycoprotein Enrichment and Mass Spectrometry

For glycoprotein enrichment 3 mg total protein from total heart lysates was incubated with either Con A or PNA coupled agarose beads at 4°C overnight, washed with lysis buffer (20 mM Tris, 150 mM NaCl, 1% (v/v) TritonX-100, complete protease inhibitor and complete phosphatase inhibitor (Roche)) and glycoproteins were eluted with 200 mM glycine buffer pH 2.5. After elution 1 M Tris buffer pH 10.4 was added and samples were stored at −20°C.

## Sample Preparation for Proteomics Analysis

For proteomics analysis, samples were sonicated (Bioruptor Plus, Diagenode, Belgium) for 10 cycles (30 s ON/60 s OFF) at high setting, at 20°C, followed by boiling at 95°C for 5 min. Reduction was followed by alkylation with 20 mM iodoacetamide (IAA, final concentration 15 mM) for 30 min at room temperature in the dark. Protein amounts were estimated, following an SDS-PAGE gel of 10 µL of each sample against an in-house cell lysate of known quantity. 30 µg of each sample was taken along for digestion. Proteins were precipitated overnight at −20°C after addition of 4 × volume of ice-cold acetone. The following day, the samples were centrifuged at 20,800 g for 30 min at 4°C and the supernatant was carefully removed (Eppendorf 5810R, Eppendorf AG, Germany). Pellets were washed twice with 300 µL ice-cold 80% (v/v) acetone in water then centrifuged at 20,800 g at 4°C for 10 min. After removing the acetone, pellets were air-dried before addition of 25 µL of digestion buffer (1 M Guanidine, 100 mM HEPES, pH 8). Samples were resuspended with sonication as explained above, then LysC (Wako) was added at 1:100 (w/w) enzyme:protein ratio and digestion proceeded for 4 h at 37°C under shaking (1,000 rpm for 1 h, then 650 rpm). Samples were then diluted 1:1 with MilliQ water and trypsin (Promega) added at 1:100 (w/w) enzyme:protein ratio. Samples were further digested overnight at 37°C under shaking (650 rpm). The day after, digests were acidified by the addition of TFA to a final concentration of 10% (v/v), heated at 37°C and then desalted with Waters Oasis^®^ HLB µElution Plate 30 µm (Waters Corporation, MA, United States) under a soft vacuum following the manufacturer´s instruction. Briefly, the columns were conditioned with 3 × 100 µL solvent B (80% (v/v) acetonitrile; 0.05% (v/v) formic acid) and equilibrated with 3 × 100 µL solvent A (0.05% (v/v) formic acid in Milli-Q water). The samples were loaded, washed three times with 100 µL solvent A, and then eluted into 0.2 ml PCR tubes with 50 µL solvent B. The eluates were dried down using a speed vacuum centrifuge (Eppendorf Concentrator Plus, Eppendorf AG, Germany). Dried samples were stored at −20°C until analysis. LC-MS data independent analysis (DIA).

Prior to analysis, samples were reconstituted in MS Buffer (5% acetonitrile, 95% Milli-Q water, with 0.1% formic acid) and spiked with iRT peptides (Biognosys, Switzerland). Peptides were separated in trap/elute mode using the nanoAcquity MClass Ultra-High Performance Liquid Chromatography system (Waters, Waters Corporation, Milford, MA, United States) equipped with a trapping (nanoAcquity Symmetry C18, 5 μm, 180 μm × 20 mm) and an analytical column (nanoAcquity BEH C18, 1.7 μm, 75 μm × 250 mm). Solvent A was water and 0.1% formic acid, and solvent B was acetonitrile and 0.1% formic acid. 1 µL of the sample (∼μg) were loaded with a constant flow of solvent A at 5 μL/min onto the trapping column. Trapping time was 6 min. Peptides were eluted via the analytical column with a constant flow of 0.3 μL/min. During the elution, the percentage of solvent B increased in a non-linear fashion from 0 to 40% in 90 min. Total run time was 115 min. The LC was coupled to the Orbitrap Exploris 480 (Thermo Fisher Scientific, Bremen, Germany) using the Proxeon nanospray source. Peptides were introduced into the mass spectrometer via a Pico-Tip Emitter 360-μm outer diameter × 20 μm inner diameter, 10 μm tip (New Objective) heated at 300°C, and a spray voltage of 2.2 kV was applied. The capillary temperature was set at 300°C. The radio frequency ion funnel was set to 30%. For DIA data acquisition, full scan mass spectrometry (MS) spectra with mass range 350–1,650 m*/z* were acquired in profile mode in the Orbitrap with resolution of 120,000 FWHM. The default charge state was set to 3^+^. The filling time was set at maximum of 60 ms with limitation of 3 × 10^6^ ions. DIA scans were acquired with 30 mass window segments of differing widths across the MS1 mass range. Higher collisional dissociation fragmentation (stepped normalized collision energy; 25.5, 27, and 30%) was applied and MS/MS spectra were acquired with a resolution of 30,000 FWHM with a fixed first mass of 200 m*/z* after accumulation of 3 × 10^6^ ions or after filling time of 47 ms (whichever occurred first). Data were acquired in profile mode. For data acquisition and processing of the raw data, Xcalibur 4.4 (Thermo Fisher) and Orbitrap Exploris 480 Tune version 2.0 were used.

## Data Processing

DIA raw data were analyzed using the directDIA pipeline in Spectronaut (v.13, Biognosysis, Switzerland). The data were searched against a species specific (Mus Muculus, 16.747 entries) and a contaminants (247 entries) Swissprot database. The data were searched with the following modifications: Carbamidomethyl (C) (Fixed) and Oxidation (M), Acetyl (Protein N-term). A maximum of two missed cleavages for trypsin and five variable modifications were allowed. Identifications were filtered to satisfy FDR of 1% on peptide and protein level. Relative quantification was performed in Spectronaut for each paired comparison using the replicate samples from each condition. The data (candidate table) and data reports (protein quantities) were then exported and further data analyses and visualization were performed with Rstudio using in-house pipelines and scripts. To select significant proteins, a log_2_FC cutoff of 0.58 and a q-value<0.05 were defined.

GSEA analysis was performed and reactome pictures were created using the online tool Webgestalt (FDR<0.05). Canonical Pathway Analysis was performed with Ingenuity Pathway Analysis software (IPA25) with all proteins in the dataset used as a custom reference set. *p*-values were corrected for multiple testing using the Benjamini-Hochberg method. Heatmaps for differential protein expression have been calculated with R version 4.0.3. Specifically, average log2 expression ratios for the three age groups comparisons (12 vs. 3 M, 22 vs. 12 M, and 22 vs. 3 M) were ranked by their respective q-values. The heatmaps (pheatmap, version 1.0.12) display the top 30 proteins and the top 30 glycoproteins. Glycoproteins were annotated using the Uniprot database (release 2020_06) and R’s UniprotR package (version 2.0.3).

## Statistical Analysis

For statistical analysis, raw data were analyzed for normal distribution with the Kolmogorov-Smirnov goodness-of-fit test or with graphical analysis using the Box-Plot and QQ-Plot. If appropriate we either used 1-way ANOVA, 2-way ANOVA or Student’s *t*-test. * indicates *p* < 0.05, **p < 0.01 and ***p < 0.0005. For statistical analysis, we used Graphpad prism 5. For all data, means with standard error of the mean (SEM) or individual data points with SEM are shown.

## Data and Software Availability

Glycome data are presented in Supplementary Table S1. The mass spectrometry proteomics data have been deposited to the ProteomeXchange Consortium via the PRIDE (Perez-Riverol et al., 2019) partner repository with the dataset identifier PXD023740.

**Project Name:** Altered glycosylation in the aging heart
**Project accession:** PXD023740
**Username:** reviewer_pxd023740@ebi.ac.uk
**Password:** uo7zIkWU
**Weblink:** https://www.ebi.ac.uk/pride/archive/projects/PXD023740/private

## Results

### Increased Cardiomyocyte Diameter and Increased Fibrosis in the Aged Mouse Heart

**FIGURE 1:**
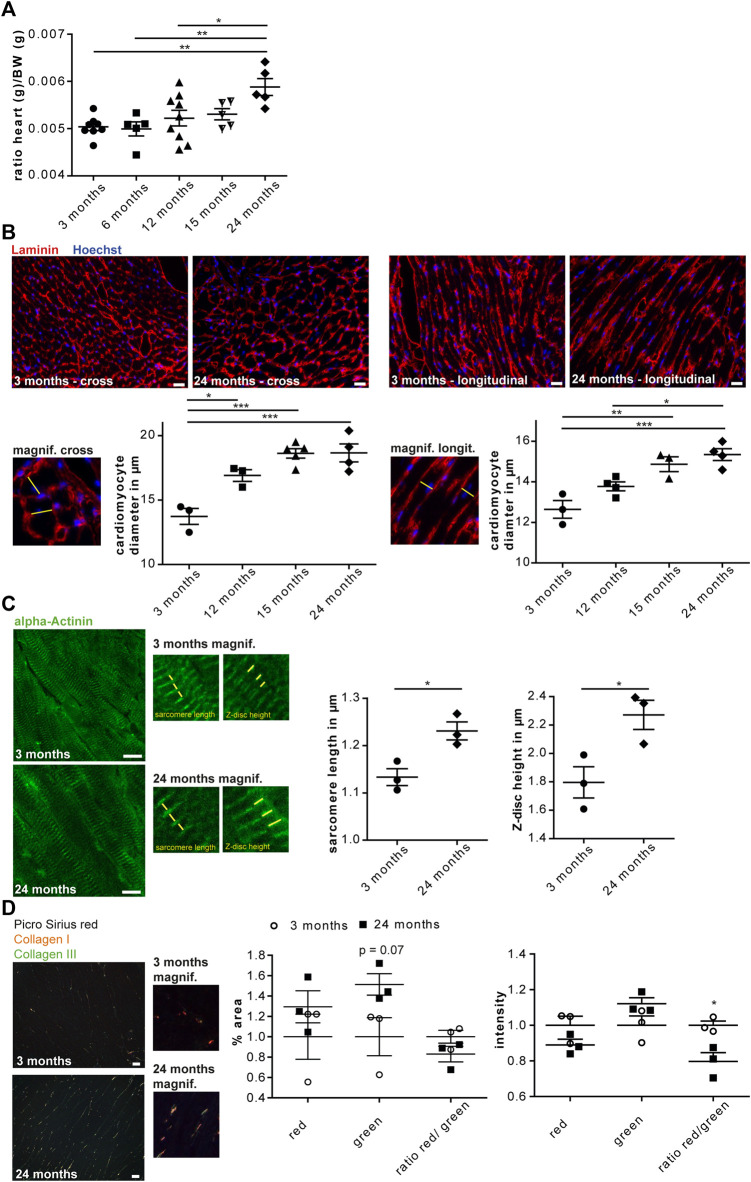
Cardiac aging in male C57BL/6J mice. **(A)** The heart/body weight ratio increases during aging (*n* = 5–9 mice per age; 1-way ANOVA with Bonferroni posthoc test). **(B)** Representative images of either cross **(left)** or longitudinal **(right)** heart sections of a 3 and 24-month-old mouse are shown (scale bar: 20 µm). Left panel: Higher magnification of cross-sectioned cardiomyocytes. An exemplary cardiomyocyte diameter is indicated (yellow line). The mean cell diameter of cross-sectioned cardiomyocytes increases with age (*n* = 3-5 mice per age, *N* = 300 cells per mouse, 1-way ANOVA with Bonferroni posthoc test). Right panel: Higher magnification of longitudinally sectioned cardiomyocytes. An exemplary cardiomyocyte diameter is indicated (yellow line). The mean cell diameter of longitudinal-sectioned cardiomyocytes increases with age (*n* = 3–5 mice per age, *N* = 200 cells per mouse, 1-way ANOVA with Bonferroni posthoc test). **(C)** Representative images of heart sections of 3 and 24-month-old mice stained for α-actinin (scale bar: 10 µm). Sarcomere length and Z-disc height are shown in higher magnification images. Sarcomere length as well as α-actinin height increase with aging (*n* = 3 mice per age, *N* = 9–11 pictures per mouse with at least 60 measurements, Student’s *t*-test). **(D)** Representative images of Picro sirius red stained heart sections of 3 and 24-month-old mouse are shown (scale bar: 25 µm). Cardiac collagen deposits increase during aging (*n* = 3 mice per age, *N* = 15 fields per mouse, 1-way ANOVA with Bonferroni posthoc test). Data are presented as individual data points with SEM.

To assess whether aging entails changes of the cardiac glycoproteome we used the C57BL/6 J mouse strain, which is a common model organism in aging research. In accordance to previous reports (Kiper et al., 2013), the heart weight increased from young (3 and 6-month-old), middle-aged (12 and 15-month-old) and old (24-month-old) mice (Figure 1A). To address whether the increase of heart weight correlates with an increase of the mean cardiomyocyte diameter, we stained heart sections for the ECM protein laminin and measured the mean cardiomyocyte diameter of cross as well as longitudinal heart sections. Both analyses suggested that the mean cardiomyocyte diameter increased between young (3-month-old) and middle-aged (12-month-old) mice and rose further up to 2 years of age (Figure 1B). These data are in agreement with previous studies reporting that hypertrophy is reflected by an increase in cardiomyocyte size and not in cell number (Senyo et al., 2013). The increase in cardiomyocyte diameter was associated with an increase in sarcomere length and Z-disc height (Figure 1C).

Picro Sirius red staining of heart sections from mice at 3 or 24 months of age showed an increase in collagen fibers and a reduced ratio of collagen type I to collagen type III fibers in 24-month-old hearts (Figure 1D). This is in agreement with ECM remodeling during cardiac aging (Burgess et al., 2001).

In summary, our data confirm previous reports on age-associated cardiac remodeling in C57BL/6J mice.

### Age Dependent Changes in High-Mannose *N*-Glycans in the Heart

**FIGURE 2:**
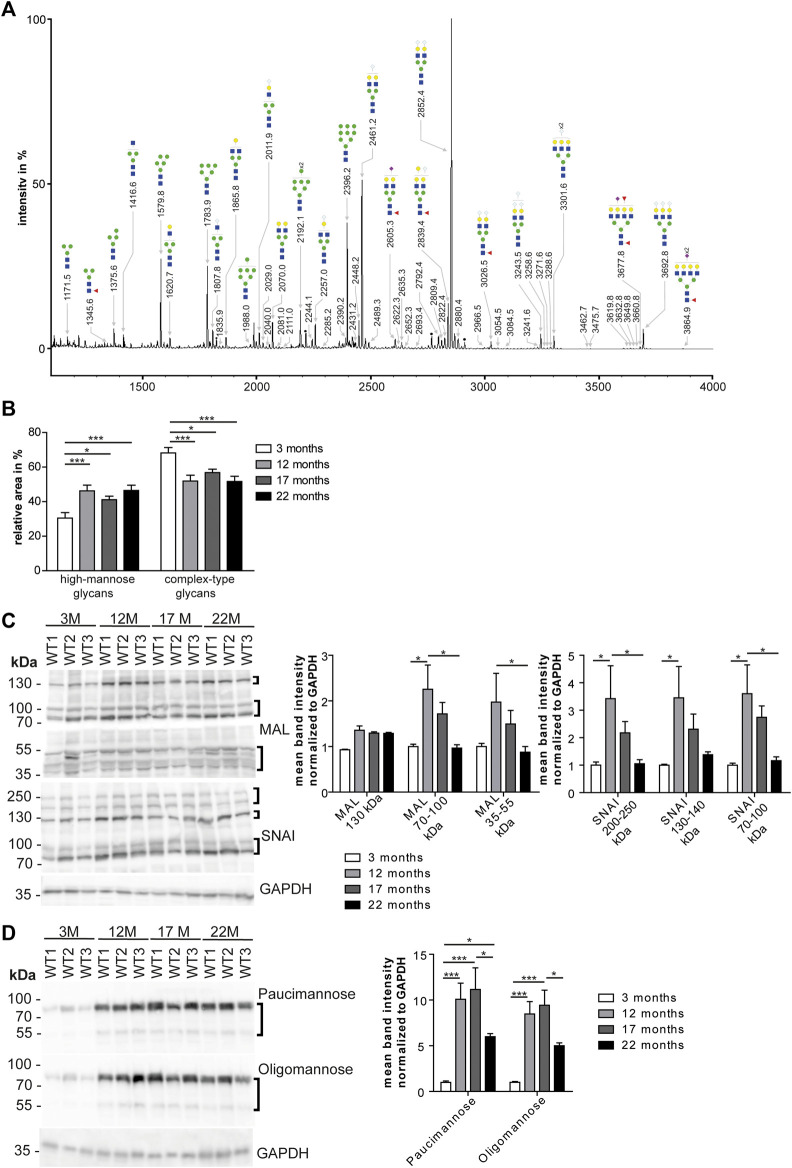
Age dependent changes of high mannose *N*-glycans in the heart. **(A)** Representative MALDI-TOF spectra of permethylated *N*-glycans measured in the positive ionization mode. All molecular ions are present in sodiated form [M + Na]^+^. Green circle: Man, yellow circle: Gal; blue square: GlcNAc, white diamond: NeuGc, violet diamond: NeuAc, red triangle: Fuc, black asterisk: non-carbohydrate contamination. **(B)** Relative intensities of the MALDI-TOF spectra from permethylated *N*-glycans released from mouse heart tissues (*n* = 6 mice per age; 1-way ANOVA with Bonferroni posthoc test). Relative intensities of individual spectra are presented in Supplementary Table S1. **(C)** Western Blots probed with lectins recognizing neuraminic acid. For quantification GAPDH served as loading control (*n* = 6 mice per age; 2-way ANOVA with Bonferroni posthoc test). MAL: Neu5(Ac/Gc)-α(2,3)-Gal-β(1,4)-GlcNAc-R, SNA: Neu5(Ac/Gc)-α(2,6)-Gal-β(1,4)-GlcNAc-R. **(D)** Western Blots incubated with antibodies recognizing mannose. For quantification GAPDH served as loading control (*n* = 6 mice per age; 2-way ANOVA with Bonferroni posthoc test). Oligomannose: 6–9 terminal mannose residues. Paucimannose: 3 terminal mannose residues. Black brackets indicate measured bands at indicated molecular weights. Quantitative data are presented as mean ± SEM. Individual data points are shown in Supplementary Figure S1.

To assess whether the glycosylation of cardiac proteins changes during aging, we released *N*-glycans from denatured glycoproteins stemming from total heart tissue lysates from 3, 12, 17, and 22-month-old mice. After purification, *N*-glycans were permethylated and analyzed by MALDI-TOF mass spectrometry. We could identify 69 *N*-glycan structures (Figure 2A; Supplementary Table S1) that were of high-mannose, hybrid and complex-type. Sialylated *N*-glycan structures contained mostly *N*-glycolylneuraminic acid but *N*-acetyl neuraminic acid was found as well (Supplementary Figures S1A, S2A; Supplementary Table S1). Relative intensities were compared for the different age groups. We observed a significant increase in high-mannose *N*-glycans (Figure 2B; Supplementary Figure S1A) in older mice (22 months) compared to young mice (3 months). (Figures 2A,B; Supplementary Figure S1A). Moreover, we detected a progressive decrease in complex-type glycans carrying terminal neuraminic acid (Supplementary Figure S1A) as well as a decrease of fucose (Supplementary Figure S1A).

Based on these data, we separated cardiac proteins by SDS polyacrylamide gel electrophoresis and probed the blots with lectins to detect specific carbohydrates of glycoproteins. α(2,3)-linked *N*-acetylneuraminic acid and/or *N*-glycolyl-neuraminic acid on β1,4-linked galactose-linked *N*-acetylglucosamine residues (Neu5Ac/Gc-α(2,3)-Gal-β(1,4)-GlcNAc-R) were detected with the maackia amurensis lectin (MAL) (Figure 2C; Supplementary Figure S1E). For the detection of α2,6-linked *N*-acetylneuraminic acid and/or *N*-glycolylneuraminic acid on β1,4-linked galactose-linked *N*-acetylglucosamine residues (Neu5Ac/Gc-α(2,6)-Gal-β(1,4)-GlcNAc-R) we used the sambucus nigra lectin (SNAI) (Figure 2C; Supplementary Figure S1E). While we observed an increase of signal intensities from 3 to 12-month-old mice, intensities decreased from 12 to 17 and 22 months of age (Figure 2C; Supplementary Figure S1E). Of note, with MAL we detected an increase in the band intensity at 130 kDa between 3 and 12 months of age that did not decrease at older age (Figure 2C; Supplementary Figure S1E). Detection of glycoproteins with lectins recognizing *N*-acetylglucosamine (WGA: binding to *N*-acetylglucosamine (GlcNAc) and *N*-acetylneuraminic acid (Neu5Ac) and/or *N*-glycolylneuraminic acid (Neu5Gc)) or terminal galactose (RCAI: terminal β1,4-linked galactose (Gal-β(1,4); PNA: β1,3-linked galactose on *N*-acetylgalactosamine residues) showed an increase of band signal intensities between 3 and 12 months of age mice which declined thereafter (Supplementary Figures S1D,E).

Immunoblot analysis with antibodies directed against paucimannose, which binds to three terminal mannose residues, or high-mannose, which detects 6–9 terminal mannose residues, revealed a drastic increase in the band intensity in 12-month-old mice, which declined slightly between 12 and 22 months of age (Figure 2D; Supplementary Figures S1F,E).

In conclusion, the most prominent finding of our mass spectrometry and Western Blot analysis suggests an age-associated increase of mannose in glycoproteins and a relative decrease of complex-type carbohydrates.

### Age-dependent Changes in Serum Mannose Concentrations and GDP-Mannose-Pyrophosphorylase-B Abundance in the Heart

**FIGURE 3:**
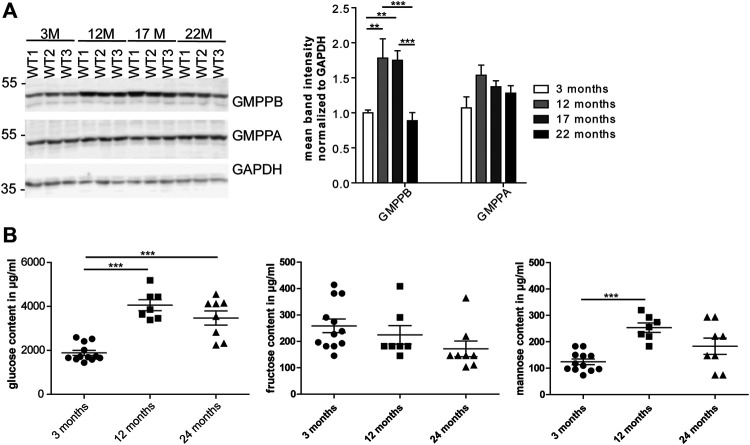
Age-dependent changes in cardiac GMPPB abundance and serum mannose concentrations. **(A)** GMPPB abundance increases between 3 and 12 months of age, while GMPPA abundance does not change. GAPDH served as loading control (*n* = 6 mice per age; 2-way ANOVA with Bonferroni posthoc test). **(B)** Sugar concentrations in serum of non-fasted mice. Glucose and mannose concentration increase between 3 and 12 months of age, while fructose concentration does not change (*n* = 7–12 mice per age; 1-way ANOVA with Bonferroni posthoc test). Data are presented as individual data points with SEM or as mean ± SEM. Individual data points are shown in Supplementary Figure S2.

As mannosylation was the most prominent alteration during aging, we wondered whether the expression of GMPPB, the enzyme, which facilitates the production of the sugar donor GDP-mannose (Koehler et al., 2013), is regulated during aging (Figure 3A; Supplementary Figure S2A). Indeed, the abundance of GMPPB in the heart was significantly higher at 12 compared to 3 months of age. In contrast, the abundance of its non-catalytically active homolog, GMPPA, which serve as an allosteric feedback inhibitor of GMPPB (Koehler et al., 2013; Franzka et al., 2021), did not change (Figure 3A; Supplementary Figure S2A).

Mannose is easily taken up in the gastrointestinal tract and together with glucose-derived mannose as well as mannose released from glycans undergoing degradation contributes to the mannose pool used for glycoconjugate synthesis (Sharma et al., 2014). We measured the mannose serum concentrations in non-fasted mice at different ages. We also measured glucose and fructose concentrations, because glucose and fructose can be easily isomerized into mannose (Flores-Díaz et al., 1997). Notably, glucose and mannose concentrations in 12-month-old mice were significantly higher compared to 3-month-old mice (Figure 3B). Fructose levels did not change during aging (Figure 3B).

Because of the dramatic increase in mannosylation and increased systemic mannose concentrations, we wondered whether a mannose-free diet would decrease serum mannose concentrations and the incorporation of mannose into glycans. Therefore, we fed mice either with normal food or mannose-free food starting at postnatal day (P) 14 up to 9 or 12 months of age and analyzed serum sugar concentrations at 9 months of age as well as the glycosylation pattern by Western Blot analysis at 12 months of age (Supplementary Figure S3). The diet, however, neither affected serum mannose concentrations nor protein glycosylation in the heart (Supplementary Figure S3).

In summary, our data show age dependent changes in serum mannose concentrations and GMPPB abundance.

### Quantitative Changes in the Cardiac Glycoproteome During Aging

**FIGURE 4:**
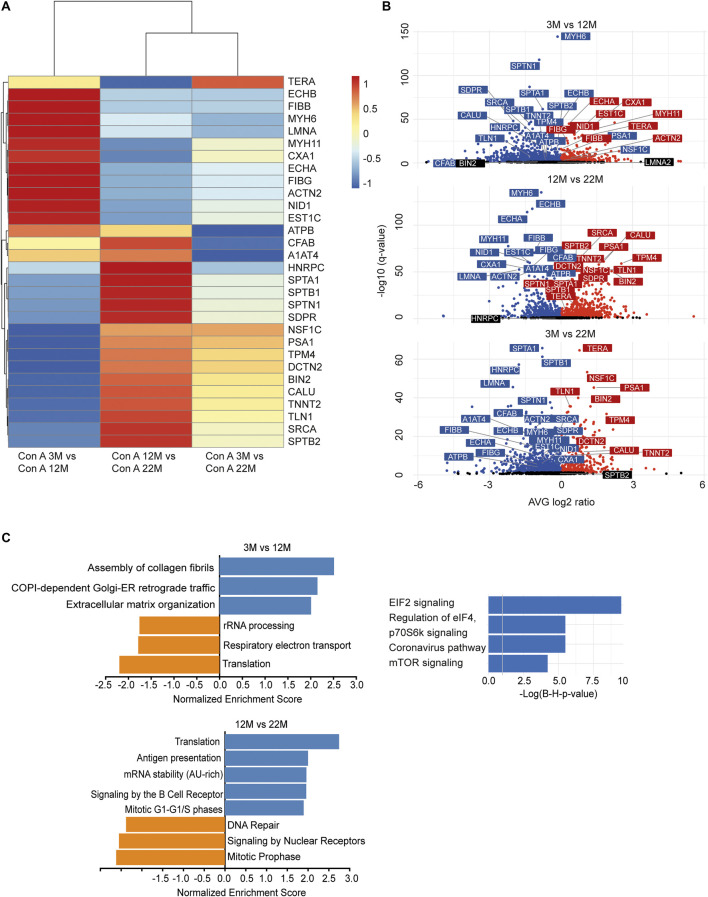
Quantitative changes in the cardiac glycoproteome during aging upon Con A pull-down. **(A)** Mass spectrometry analysis of top 30 cardiac muscle proteins of aging male mice after Con A pull-down. Proteins significantly up-regulated/or hyperglycosylated between two different ages are shown in red and those down-regulated/or hypoglycosylated in blue (*n* = 3 mice per age). **(B)** Volcano plots for all identified proteins after Con A enrichment with a q-value below 0.05. Proteins significantly up-regulated/or hyperglycosylated between two different ages are shown in red and those down-regulated/or hypoglycosylated in blue, while unchanged proteins are shown in black (*n* = 3 mice per age). **(C)** Affected pathways are shown for Con A (glycans carrying mannose) enriched proteins. Gene set expression analysis (GSEA) showing reactome with a false-discovery rate (FDR) of <0.05. Up-regulated/or glycosylated GSEA reactome pathways are shown in blue, down-regulated/or -glycosylated GSEA reactome pathways are indicated in orange. Most affected pathways identified by ingenuity pathway analysis (IPA) (FDR <0.1).

**FIGURE 5:**
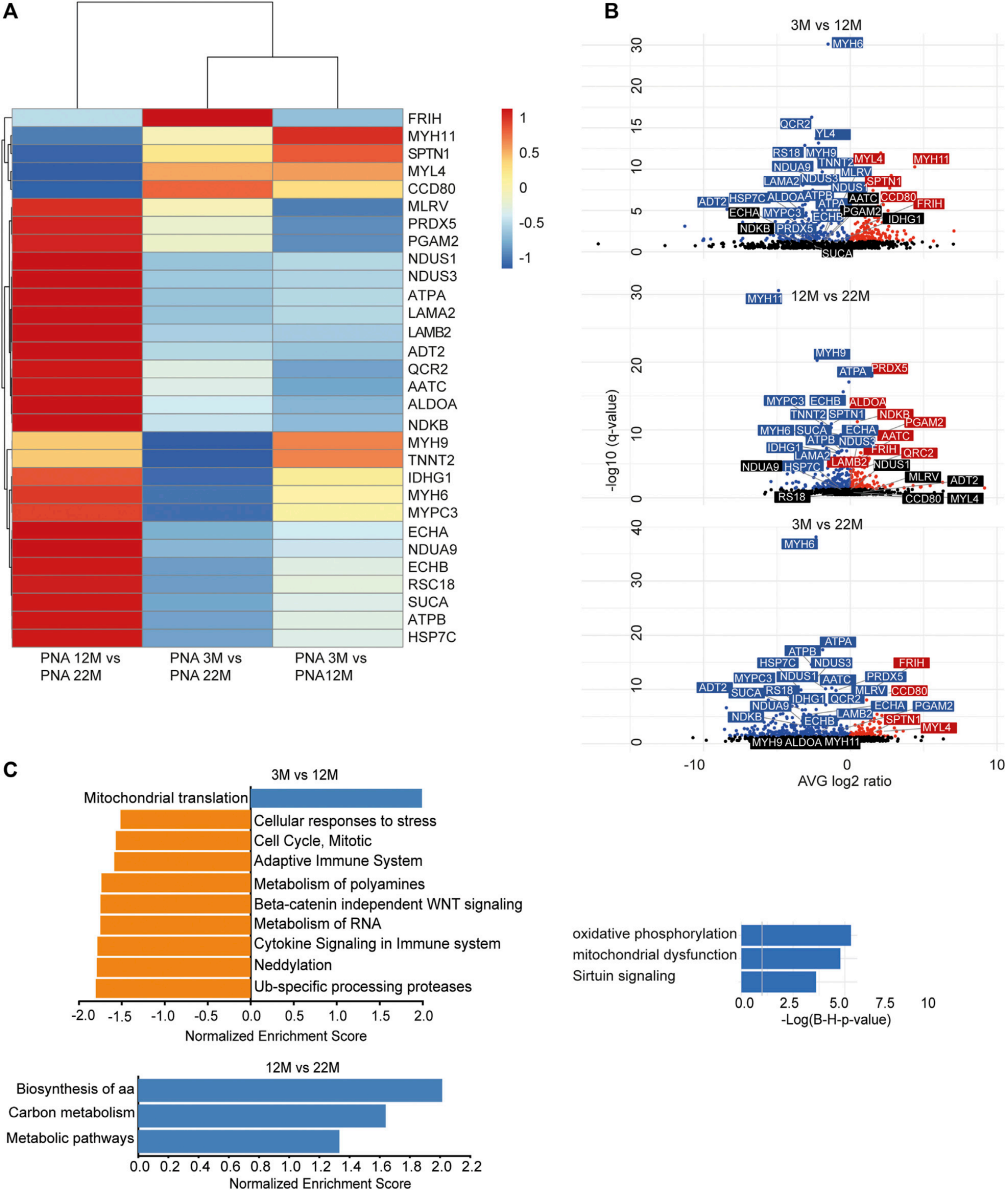
Quantitative changes in the cardiac glycoproteome during aging upon PNA pull-down. **(A)** Mass spectrometry analysis of top 30 cardiac muscle proteins of aging male mice after PNA pull-down. Proteins significantly up-regulated/or hyperglycosylated between two different ages are shown in red and those down-regulated/or hypoglycosylated in blue (*n* = 3 mice per age). **(B)** Volcano plots for all identified proteins after PNA enrichment with a q-value below 0.05. Proteins significantly up-regulated/or hyperglycosylated between two different ages are shown in red and those down-regulated/or hypoglycosylated in blue, while unchanged proteins are shown in black (*n* = 3 mice per age). **(C)** Affected pathways are shown for PNA (glycans carrying terminal galactose) enriched proteins. Gene set expression analysis (GSEA) showing reactome with a false-discovery rate (FDR) of <0.05. Up-regulated/or glycosylated GSEA reactome pathways are shown in blue, down-regulated/or -glycosylated GSEA reactome pathways are indicated in orange. Most affected pathways identified by ingenuity pathway analysis (IPA) (FDR <0.1).

As we found an increased incorporation of galactose (Supplementary Figure S1C) as well as mannose residues (Figure 2D) in glycans, we performed a Con A (for enrichment of proteins carrying mannose) or PNA pull-down (to enrich proteins carrying terminal galactose) of heart protein lysates from 3, 12 and 22-month-old mice in order to identify age-dependent glycoprotein alterations. Subsequently, the recovered proteins were identified and relatively quantified by mass spectrometry (Figures 4, 5; Supplementary Figure S4). In total, we found ∼1,670 proteins upon Con A pull-down of which ∼480 proteins were significantly up- or down-regulated/or differentially glycosylated between young, middle-aged and old mice. Upon PNA pull-down we identified ∼900 proteins of which ∼80 proteins were significantly up- or down-regulated/or differentially glycosylated (Qvalue≤0.05, no. unique peptides≥2, AVG. log2ratio≥0.58 or ≤ −0.58). Comparison between samples from 3, 12 and 22-month-old mice revealed that glycoproteins, which were more abundant in middle-aged compared to young mice, often decreased in aged mice (Figures 4A,B, 5A,B; Supplementary Figure S4). The pathways “RNA processing” and “translation”, “ECM organization”, “cell cycle”, “respiratory electron transport”, and “immune system” were most prominently regulated between different ages after Con A pull-down (Figure 4C). For proteins pulled down with PNA the most prominently regulated pathways were “transcription”, “cell cycle”, “DNA repair”, “respiratory electron transport”, “sirtuin signaling”, and “oxidative phosphorylation” (Figure 5C).

### Altered Expression of Proteins Necessary for Cell Stabilization and Glycoprotein Quality Control in Aged Hearts

**FIGURE 6:**
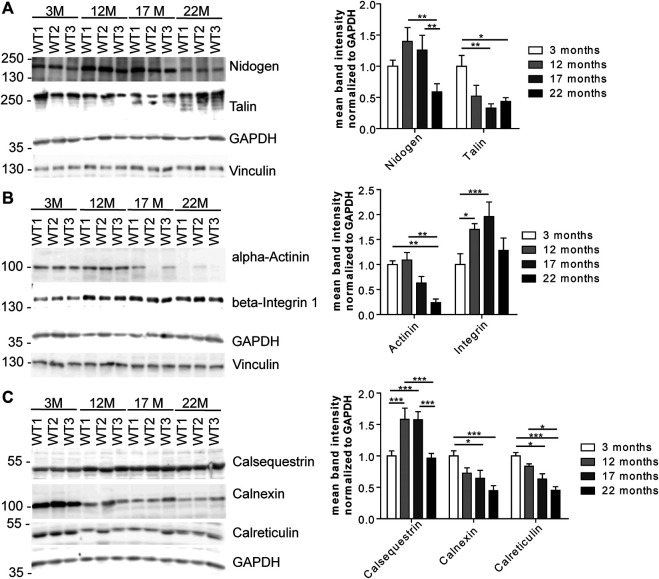
Expression of candidate proteins involved in stabilization, organization, signaling and stress responses identified in aging C57BL/6J male hearts. **(A)** Western Blot analysis of candidate glycoproteins identified by mass spectrometry after Con A pull-down. GAPDH and vinculin served as loading control (*n* = 6 mice per age; 2-way ANOVA with Bonferroni posthoc test). **(B)** Western blot analysis of the candidate protein alpha-actinin and the talin-interacting protein beta-integrin 1. GAPDH and vinculin served as loading control (*n* = 6 mice per age; 2-way ANOVA with Bonferroni posthoc test). **(C)** Western Blot analysis of calcium-binding ER proteins selected after mass spectrometry of lectin pull-down. GAPDH served as loading control (*n* = 6 mice per age; 2-way ANOVA with Bonferroni posthoc test). Quantitative data are presented as mean ± SEM. Individual data points are shown in Supplementary Figure S2.

We wondered whether the changes in the abundance of glycoproteins either reflect changes in the efficiency of proteins to be pulled down because of changes of their glycosylation or rather changes in the overall protein abundance. Therefore, we selected some candidate glycoproteins involved in 1) ECM remodeling and signaling, 2) cytoskeleton and cell cycle organization and/or 3) ER stress response for Western blot analysis (Figure 6) as we identified several proteins related to these pathways (Figures 4, 5; Supplementary Figure S4, PRIDE: PXD023740). After immunodetection, we quantified the respective protein bands (representative immunoblots are shown in Figure 6). Either GAPDH or Coomassie stained membranes served as loading control for normalization. Band intensities of (glyco)-proteins of a high molecular weight were additionally normalized to vinculin. Since these different approaches lead to comparable results, we only present the normalization to GAPDH, and, in case for high molecular proteins, to vinculin too.

Immunoblot analysis of the ECM-glycoprotein nidogen revealed an increase in nidogen abundance between young and middle-aged mice that decreased between 17 and 22 months of age. No obvious shift toward a higher or lower molecular weight was detected as would be expected for major changes in protein glycosylation (Figure 6A; Supplementary Figure S2B). In contrast, the abundance of the *O*-glycosylated protein talin, which connects membrane-bound beta-integrin and the subcortical actin-α-actinin network (Martel et al., 2000), progressively decreased during aging as judged from immunoblot analysis. Additional bands at older ages may indicate that the glycosylation of talin might change with aging (Figure 6A; Supplementary Figure S2B). The abundance of alpha-actinin decreased while that of beta-integrin increased with age (Figure 6B; Supplementary Figure S2C). Moreover, beta-integrin shifted toward a slightly higher molecular weight, which may indicate increased glycosylation (Figure 6B; Supplementary Figure S2C).

Notably, we found several ER-related calcium binding proteins to be regulated with age (Figures 4A,B, 5A,B; Supplementary Figure S4, e.g., calumenin). Calsequestrin is a mannosylated calcium-storage protein of the sarcoplasmic reticulum (SR) and changes in calsequestrin glycosylation have been associated with heart failure, previously (Kiarash et al., 2004; Sanchez et al., 2012). Immunoblot analysis for calsequestrin suggested that its abundance increases with age. Moreover, we observed a clear shift toward a higher molecular weight (Figure 6C; Supplementary Figure S2D) which may indicate incorporation of additional mono or oligosaccharides into its glycochains. The abundance of calnexin and calreticulin, which are calcium-binding chaperones involved in ER quality control of forming glycoproteins (Williams, 2006), drastically decreased with aging (Figure 6C; Supplementary Figure S2D). In addition, calreticulin bands shifted toward a slightly higher molecular weight during aging (Figure 6C; Supplementary Figure S2D).

Taken together, our data suggest alterations in both the expression and the glycosylation status of glycoproteins during aging.

## Discussion

Glycosylation has an important impact on physical and functional properties of proteins. Therefore, different glycosylation patterns result in structural and functional diversification of a single protein to yield a set of glycosylation variants. Glycosylation changes during aging have been shown over 20 years ago (Parekh et al., 1988) and have also been replicated in large population studies (Knezevic et al., 2010; Vanhooren et al., 2010; Pucić et al., 2011; Ruhaak et al., 2011). Most of these studies addressed plasma glycoproteins (Parekh et al., 1988; Knezevic et al., 2010; Vanhooren et al., 2010; Pucić et al., 2011; Ruhaak et al., 2011). Studies on cardiac glycoproteins mainly addressed changes in glycosylation in the diseased heart (Fülöp et al., 2007; Montpetit et al., 2009; Deng et al., 2016; Nagai-Okatani and Minamino, 2016; Marques-Da-Silva et al., 2017). Only one study addressed glycosylation in the aging aortic valve (Przybyło et al., 2007).

Here, we set out to analyze whether the glycoproteome of the heart changes during aging and may thus contribute to cardiac aging. Our analysis of heart tissue clearly demonstrate an increase in glycans carrying mannose residues during aging by Western Blot analyses as well as MALDI-TOF based glycome analysis. We grouped *N*-glycans into either high-mannose (Man_3_GlcNAc_2_ up to Man_9_GlcNAc_2_), complex-type or hybrid-type glycans according to the regular glycan classification (Varki et al., 2009) and sub-grouped complex-type structures according to fucosylation and sialylation. We found a raise in the absolute amount of glycans with terminal galactose and terminal neuraminic acid on β1,4-linked galactose-linked *N*-acetylglucosamine residues between young and middle-aged mice that decreased at old age. As reported for human plasma glycoproteins (Miura and Endo, 2016), the relative intensity of complex-type *N*-glycans decreased during aging. Taken together, we can conclude that either a higher proportion of glycoproteins carries high-mannoses or that complex-branched *N*-glycan structures are replaced by mannose in *N*-glycans of the murine heart. This is in agreement with a previous study addressing age-related changes in the glycoproteome of aortic valves (Przybyło et al., 2007). This study included postmortem hearts of individuals with no history of cardiac disease with a maximal age of 54 years (Przybyło et al., 2007). In agreement with this study, we found an increase of high-mannose glycans and an increase of signal intensities for SNAI probed Western Blots from 3 and 12-month-old mice. Other studies including samples from patients suffering from cardiomyopathy reported a reduction of sialylation of cardiac glycoproteins (Deng et al., 2016). Together with our data that Neu5Ac/Gc-Gal-GlcNAc decreases in 22-month-old mice and our MS data showing a reduction of complex-type glycans carrying terminal neuraminic acid, this suggests that sialylation might be critical for heart function. Notably, the reduction of complex-type *N*-glycans in our MS analysis of aged murine hearts corresponds with findings for mannosyl (α-1,3-)-glycoprotein β-1,2-*N*-acetylglucosaminyltransferase (Mgat1) KO mice, which develop dilated cardiomyopathy (Ednie et al., 2019).

Our data are consistent with an increase in mannosylation of plasma and serum proteins from young, old and centenarian females (Miura et al., 2018). In contrast to our findings, however, this study found an increase in core fucose and highly sialylated structures during aging, while less sialylated structures such as NeuAc_2_Hex_5_HexNAc_4_ were decreased (Miura et al., 2018). We detected a decrease in complex-type glycans carrying terminal neuraminic acid such as NeuAc_2_Hex_5_HexNAc_4_, as well as fucosylated glycans in mass spectrometry and an increase of fucosylated glycans between 12 and 17-month-old mice. It should be noted that the low abundance of fucosylated stuctures did not allow investigating the fucose linkage in details. Organ specificity, species, gender, nutrition and/or environmental factors may account for these discrepancies.

It has been reported that glucose levels increase during aging due to influences by body fat and physical fitness, especially between young and middle-aged people (Shimokata et al., 1991; Muller et al., 1996; Elahi and Muller, 2000). As the increased incorporation of mannose residues into glycan structures was the most prominent change during aging, we wondered if serum sugar concentrations might be altered. Remarkably, we found an increase of the mannose concentration in the blood of aged mice. Mannose is easily taken up in the gastrointestinal tract and together with glucose-derived mannose as well as mannose released from degraded glycans contributes to the mannose pool used for glycoconjugate synthesis. The increase in free mannose levels in aged mice may reflect increased release from glycans and/or increased generation of mannose from glucose. Notably, dietary mannose supplementation partially rescued the hypoglycosylation due to defects in phosphomannomutase 2 (PMM2), which converts mannose-6-phosphate to mannose-1-phosphate, or phosphomannose isomerase (PMI), which facilitates the interconversion of fructose 6-phosphate and mannose-6-phosphate (Schneider et al., 2011). We thus wondered whether dietary mannose restriction may have consequences for the glycosylation status of the heart. To this end, we either fed mice with a normal diet or a diet, in which mannose was replaced by sucrose, starting upon postnatal day 14 for almost 12 months and analyzed the glycosylation profile. Of note, we did not detect any obvious effect of the diet on serum mannose concentrations or the cardiac glycome. This may suggest that most of the free serum mannose comes from glycan trimming and glycoprotein degradation.

Because of the increased incorporation of mannose residues into cardiac glycoproteins at 12 months of age as well as increased serum mannose levels, we wondered whether the expression of the enzyme GMPPB, which facilitates the production of the sugar donor GDP-mannose, may be regulated. We also tested the levels of its non-catalytic homolog GMPPA, which serves as an allosteric feedback inhibitor of GMPPB, because GMPPA loss-of-function mutations result in increased GDP-mannose levels (Koehler et al., 2013; Franzka et al., 2021). In agreement with increased incorporation of mannose into glycans, the abundance of GMPPB was increased at 12 months of age, while the abundance of GMPPA was not. These findings are in agreement with previous studies reporting that the activity of enzymes necessary for glycosylation, such as sialyltransferase, increases during aging (Allalouf et al., 1988). Our data thus suggest that changes in alterations in the glycosylation machinery contribute to age-related changes of the glycoproteome.

Since we found an increase in high-mannose glycans in the MALDI-TOF based glycome analysis of total heart lysates, we performed a lectin pull-down prior to (glyco-)-protein identification by LC-MS. Of note, in Con A as well as PNA pull-downs we found alterations in glycans of the high-mannose type, such as complement C3 and alpha-2-macroglobulin as well as alterations in glycans of the complex-type, such as serotransferrin, kininogen, and fibrinogen. All these glycoproteins with complex-type *N*-glycans have a molecular weight between 70 and 100 kDa. Alpha-2-macroglobulin has been reported to show 250, 160, 97, and 80 kDa protein bands on reduced SDS-PAGE (Riley et al., 2019). Notably, 70–100 kDa is the molecular weight where we detected the strongest signal intensities with PNA and the antibodies directed against paucimannose and oligomannose. Moreover, the relative abundance of these glycoproteins carrying either high-mannose *N*-glycans or complex-type *N*-glycans was reduced in samples of the aged cohorts. This may indicate that these glycoproteins are degraded more rapidly because of incorrect glycosylation. In fact, it has been reported that some glycoproteins decrease with aging (Itakura et al., 2016).

Interestingly, most proteins and pathways that were upregulated/or differentially glycosylated between young and middle-aged mice were down-regulated/or differentially glycosylated between middle-aged and old mice. Notably, six glycoproteins (endothelin-converting enzyme 1, integrin beta-1, neprilysin, cleft lip and palate transmembrane protein 1 homolog, transferrin receptor protein 1, stromal interaction molecule 1) obtained from the Con A pull-down that were upregulated in this study were previously found to be high-mannosylated (Riley et al., 2019).

Comparison of our lectin pull-down data with published data for the murine heart proteome (Yu et al., 2020) allowed the identification of several proteins including nidogen, laminin, ankyrin, NADH dehydrogenase, or myosin, which were synonymously regulated in both approaches. While only a few proteins (∼20 out of ∼4,400 identified proteins) were altered significantly by proteome analysis (Yu et al., 2020), ∼480 out of ∼1,670 identified proteins pulled-down with Con A were significantly regulated and ∼80 out of ∼900 proteins pulled-down with PNA. This confirms previous data that enrichment strategies help to detect ageing-related alterations (Di Sanzo et al., 2020). In agreement with the whole proteome analysis (Yu et al., 2020), our analysis also identified electron transport and inflammation to be regulated during aging. Moreover, we confirmed the regulation of known ageing-related pathways (for example sirtuin signaling, mitochondrial dysfunction, mTOR signaling (Downes et al., 1989; Burgess et al., 2001; Lakatta, 2003; Judge et al., 2005; Ouzounian et al., 2008; Senyo et al., 2013; Wang and Shah, 2015; Shimizu and Minamino, 2019)).

Both approaches also identified robust changes of ECM proteins (Burgess et al., 2001). E.g., beta-integrin levels increased at older age in agreement to previous studies (Ding et al., 2000). Moreover, beta-integrin bands shifted toward a slightly higher molecular weight which may reflect changes in glycosylation. The integrin interaction partners talin and nidogen (Zhang and Gunst, 2006; Kim et al., 2011; Klapholz and Brown, 2017) showed age-dependent protein changes, too. Moreover, we observed additional talin protein bands by immunoblot analysis suggesting altered talin glycosylation. These data point to altered structural organization of cardiac myocytes which may be relevant for signal transduction and cardiac contractility.

Because glycosylation is initiated at the ER (Williams, 2006), we also had a closer look on ER-related proteins in our screen Remarkably, we identified several calcium-binding proteins of the ER to be strongly regulated during aging. E.g., the abundance of calsequestrin, the major calcium-storage protein of the ER, increased with age in immunoblot analysis and shifted toward a slightly higher molecular weight, which may suggest increased glycosylation. Moreover, we found increased calsequestrin levels upon Con A pull-down in older mice suggesting increased mannosylation of calsequestrin during aging. It has been shown that mutations in calsequestrin can lead to additional oligosaccharides resulting in a lower calcium-binding capacity and an altered polymerization (Kirchhefer et al., 2010; Sanchez et al., 2012). In dogs with induced heart failure calsequestrin related glycans contained more mannose residues indicating less mannose trimming. Moreover, calsequestrin turnover was increased leading to lower calsequestrin levels (Kiarash et al., 2004; Sanchez et al., 2012; Jacob et al., 2013).

In agreement with previous studies (Choi and Kim, 2004), we also found a decrease of calnexin and calreticulin with age. Calreticulin bands shifted toward a slightly higher molecular weight, which may result from more complex glycan structures. This may further contribute to changes of the glycoproteome, because calnexin and calreticulin are involved in the ER quality control of forming glycoproteins (Williams, 2006). The impaired folding of proteins in the ER is a trigger for the activation of a complex signaling program, the unfolded protein response (UPR), which aims to restore the ER folding environment. In our pull-down approach, several proteins involved in the UPR, such as IRE1α-, eIF2- and mTOR-downstream signaling (e.g., COPS5, eIF2, HSPA5, OPA1, CLU, PDIA, and TMX1) were regulated during aging.

Taken together, this report is the first study showing a clear alteration in the glycosylation profile of the aging heart with mannosylation being the most prominent difference. Possibly, these changes reflect alterations of the glycosylation machinery, such as alterations in GMPPB abundance. Our MS analysis of glycoprotein pull-downs suggests changes of proteins relevant for pathways that have previously been reported to be aging relevant, such as ER stress (Li et al., 2019), sirtuin signaling (Zullo et al., 2018), ECM remodeling (Horn et al., 2012), cytoskeleton network (Amberg et al., 2012), and the immune system (Swirski and Nahrendorf, 2018).

## Data Availability

Glycome data are presented in Supplementary Table S1. The mass spectrometry proteomics data have been deposited to the ProteomeXchange Consortium via the PRIDE (Perez-Riverol et al., 2019) partner repository with the dataset identifier PXD023740.
